# Randomized Double-Blind Trial of Pregabalin Versus Placebo in Conjunction With Palliative Radiotherapy for Cancer-Induced Bone Pain

**DOI:** 10.1200/JCO.2015.63.8221

**Published:** 2015-12-07

**Authors:** Marie Fallon, Peter J. Hoskin, Lesley A. Colvin, Susan M. Fleetwood-Walker, Douglas Adamson, Anthony Byrne, Gordon D. Murray, Barry J.A. Laird

**Affiliations:** Marie Fallon and Barry J.A. Laird, Edinburgh Cancer Research Centre, University of Edinburgh; Lesley A. Colvin, Western General Hospital and University of Edinburgh; Susan M. Fleetwood-Walker, School of Biomedical Sciences, University of Edinburgh; Gordon D. Murray, Centre for Population Health Sciences, University of Edinburgh, Edinburgh; Peter J. Hoskin, Mount Vernon Hospital Cancer Centre, Middlesex, and University College London; Douglas Adamson, Princess Alexandra Centre, Ninewells Hospital, Dundee; Anthony Byrne, Marie Curie Palliative Care Research Centre, Institute of Cancer and Genetics, Cardiff University, Cardiff, United Kingdom; and Barry J.A. Laird, European Palliative Care Research Centre, Norwegian University of Science and Technology, Trondheim, Norway.

## Abstract

**Purpose:**

Cancer-induced bone pain (CIBP) affects one third of patients with cancer. Radiotherapy remains the gold-standard treatment; however, laboratory and clinical work suggest that pregabalin may be useful in treating CIBP. The aim of this study was to examine pregabalin in patients with CIBP receiving radiotherapy.

**Patients and Methods:**

A multicenter, double-blind randomized trial of pregabalin versus placebo was conducted. Eligible patients were age ≥ 18 years, had radiologically proven bone metastases, were scheduled to receive radiotherapy, and had pain scores ≥ 4 of 10 (on 0-to-10 numeric rating scale). Before radiotherapy, baseline assessments were completed, followed by random assignment. Doses of pregabalin and placebo were increased over 4 weeks. The primary end point was treatment response, defined as a reduction of ≥ 2 points in worst pain by week 4, accompanied by a stable or reduced opioid dose, compared with baseline. Secondary end points assessed average pain, interference of pain with activity, breakthrough pain, mood, quality of life, and adverse events.

**Results:**

A total of 233 patients were randomly assigned: 117 to placebo and 116 to pregabalin. The most common cancers were prostate (n = 88; 38%), breast (n = 77; 33%), and lung (n = 42; 18%). In the pregabalin arm, 45 patients (38.8%) achieved the primary end point, compared with 47 (40.2%) in the placebo arm (adjusted odds ratio, 1.07; 95% CI, 0.63 to 1.81; *P* = .816). There were no statistically significant differences in average pain, pain interference, or quality of life between arms. There were differences in mood (*P* = .031) and breakthrough pain duration (*P* = .037) between arms. Outcomes were compared at 4 weeks.

**Conclusion:**

Our findings do not support the role of pregabalin in patients with CIBP receiving radiotherapy. The role of pregabalin in CIBP with a clinical neuropathic pain component is unknown.

## INTRODUCTION

Cancer-induced bone pain (CIBP) is the most common type of cancer pain, affecting one third of patients.^1,2^ CIBP exists as a combination of background and breakthrough pain, with the latter either being related to events (eg, physical activity) or occurring spontaneously without any obvious precipitating factor.^3^ In CIBP, this poses a particular challenge. Standard analgesics (eg, opioids, nonsteroidal anti-inflammatories, bisphosphonates) may be useful in controlling background pain; however, they are often ineffective in treating breakthrough pain.^4^

Radiotherapy is the gold-standard treatment for CIBP; however, meta-analyses have demonstrated that only 25% of patients will achieve complete pain relief, whereas 41% will achieve partial pain relief, with onset of analgesia taking 4 to 6 weeks.^5^ Many patients with CIBP do not achieve acceptable levels of analgesia.

Animal models have been developed to understand the pathophysiology of CIBP.^6^ Focal bone cancer pain models are used currently, and these provide a robust model (with features similar to CIBP in humans). Through these, it has become evident that the underlying pathophysiology of CIBP differs from that in standard neuropathic or inflammatory pain models, although containing elements of both as well as involving additional factors arising from host–cancer cell interactions.^7,8^ Excitability in dorsal horn sensory processing pathways is markedly increased, resulting in both electrophysiologic and behavioral hypersensitivity.^9^ This means that painful and nonpainful peripheral nerve input (eg, touch, vibration, thermal stimuli) result in ongoing and stimulus-evoked pain that can be exacerbated by movement.

Animal models have been used to assess whether agents used for neuropathic pain could correct the abnormal dorsal horn neuronal architecture and minimize the central sensitization, which also exists in CIBP—particularly whether drugs that bind to the *α*2-*δ* subunit of calcium channels (eg, pregabalin, gabapentin) could act to reduce pain transmission. Expression of the *α*2-*δ*1 subunit in dorsal root ganglia is increased after peripheral nerve injury, where it is thought to facilitate channel trafficking to the plasma membrane and thereby contribute to development of central sensitization and pain hypersensitivity.^10-12^ Pregabalin and gabapentin have proven efficacy in a variety of chronic pain models (eg, nerve injury), probably through a reduction in excitatory neurotransmitter release and central sensitization.^13^ Animal studies have also reported efficacy in CIBP models, suggesting that pregabalin and gabapentin may be of value clinically in treating CIBP.^9^

In the clinical setting, pregabalin is being used increasingly to treat CIBP.^14-17^ This may be the result of emerging animal studies but may also be because of anecdotal findings. In palliative care, many of the drugs used in routine clinical practice have limited or no evidence supporting their use, but they are embedded in clinical practice. Therefore, it is fundamental that pregabalin be examined in a robust clinical trial within the relevant population before it becomes more widely adopted as a treatment for CIBP.

Following this translational paradigm, and given the need to improve management of CIBP and the need to assess the role of pregabalin fully in the clinical setting, a randomized double-blind trial of pregabalin (Lyrica; Pfizer, Tadworth, United Kingdom) versus placebo in conjunction with palliative radiotherapy for CIBP was conducted. Our hypothesis was that *α*2-*δ* calcium channel ligands may have a role in CIBP in humans, mirroring the preclinical findings.

## PATIENTS AND METHODS

### Trial Design and Participants

This was a multicenter, double-blind randomized trial of pregabalin versus placebo in conjunction with palliative radiotherapy for CIBP. Eligible patients were age ≥ 18 years, had radiologic evidence of metastatic bone disease, and were scheduled to receive radiotherapy for ≥ one site of clearly identifiable bone pain. Other key inclusion criteria were: life expectancy > 2 months, pain score (worst pain) ≥ 4 (on 0-to-10 numeric rating scale [NRS]) at the site of pain, scheduled to be treated with radiotherapy at that site, and ability to provide written informed consent. The main exclusion criteria were: current gabapentin or pregabalin use, significant renal impairment (creatinine clearance < 60 mL/min), and receiving wide-field irradiation. Patients who had any change in anticancer therapy before entering the trial with the potential to influence pain during the trial were excluded. Initially, radiotherapy to vertebral sites was an exclusion criterion, but this was subsequently removed as a major protocol modification, as discussed and approved by the trial steering committee, after 14 patients had been consented. The trial was conducted in five cancer centers in the United Kingdom: the Beatson West of Scotland Cancer Centre (Glasgow), Edinburgh Cancer Centre (Edinburgh), Velindre Cancer Centre (Cardiff), Princess Alexandra Tayside Cancer Centre (Dundee), and Mount Vernon Hospital (London).

### Procedures

After giving consent, patients entered a run-in phase (maximum, 2 weeks) where their analgesia was optimized before radiotherapy to ensure, where possible, there were minimal changes in analgesia after random assignment and radiotherapy and to allow the effect of the intervention (pregabalin or placebo) alone to be assessed. In such cases, patients would have stable pain; however, this would still be suboptimal, necessitating the need for radiotherapy.

Some patients had stable pain control and analgesic requirements before random assignment. Others had to have analgesics modified before random assignment. At the point of receiving radiotherapy, patients were only randomly assigned if they still met the eligibility criteria, with critically worst pain score ≥ 4 (0-to-10 NRS).

The radiotherapy regimen was either 8 Gy in one fraction or 20 Gy in five fractions, as per the decision of the patient's oncologist. Baseline assessments were performed in the 24 hours before the first fraction. Random assignment was carried out after the baseline assessments and before radiotherapy. Patients received either pregabalin or placebo and were given a 35-day supply of medication. Patients were instructed to take one capsule of the trial medication twice daily (12 hours apart). Each capsule contained either 75 mg of pregabalin or placebo. The trial medication was supplied free by Pfizer.

Patients were contacted every 2 to 3 days to encourage compliance with the medication and monitor for any adverse events. Every 7 days from baseline (days 8, 15, and 22), a formal assessment of analgesia was undertaken. Where a clinically meaningful improvement in pain had not occurred (defined as ≥ 2-point decrease on 0-to-10 NRS and/or patient felt adequate analgesia had not been achieved), the dose of the trial medication was increased as follows: baseline (day 1), pregabalin or placebo 75 mg twice daily; day 8, pregabalin or placebo 150 mg twice daily; day 15, pregabalin or placebo 225 mg twice daily; and day 22, pregabalin or placebo 300 mg twice daily.

### End Points

End points were assessed at 1, 2, 3, and 4 weeks after the first fraction of radiotherapy. Analyses relate to assessments at 4 weeks unless otherwise stated.

The primary end point was improvement in CIBP at the site of radiotherapy by week 4. An improvement was a reduction of ≥ 2 points on a 0-to-10 NRS for worst pain, accompanied by a stable or reduced opioid medication dose, compared with baseline. A decrease of ≥ 2 points on a 0-to-10 NRS is accepted as a clinically meaningful improvement in pain in studies of analgesic interventions and is in keeping with guidelines on end points in clinical trials in bone metastases.^18,19^

Secondary end points included: assessment of worst and average pain, assessment of functional interference of pain in day-to-day living (using Brief Pain Inventory [BPI]),^20^ analgesic requirements, tolerability of pregabalin, global quality-of-life scores (using EuroQol thermometer),^21^ and mood (using Hospital Anxiety and Depression Scale [HADS]).^22^

### Statistical Analysis

This analysis was based on the objective of showing superiority of pregabalin versus placebo in improving CIBP using the primary end point. An intention-to-treat approach was used.

The null hypothesis tested was that there was no difference in analgesia between the trial arms. A sample size of 206 patients was planned, based on the assumption that 50% of patients in the placebo arm would improve (ie, experience ≥ 2-point drop in worst pain score against background of stable or reduced opioid dose). To detect an improvement to 70% (ie, treatment effect of 20%) in the pregabalin arm, 103 patients were required to complete each arm, using a two sided *χ*^2^ test with *α* of 0.05% and 80% power.

The primary analysis used a conservative approach, where any patient who did not complete the trial was assumed not to have achieved the primary end point of ≥ 2-point drop in worst pain score against a background of stable or reduced opioid dose. This meant that all randomly assigned patients could be included in the primary intention-to-treat analysis. However, this approach tends to attenuate any true treatment effect, so the target sample size was increased to 260. This would give 80% power at the 5% significance level to detect a more conservative treatment effect of 18%. The safety population included all patients who received any dose of the trial medication, and adverse event reporting was continued until 30 days after trial completion.

Random assignment was implemented by the Cancer Research UK Clinical Trials Unit Glasgow using a minimization algorithm with a random element, based on: fractionation regimes (single *v* multiple), cancer type (breast or prostate *v* other tumor types), and site of bone metastasis (vertebrae *v* nonvertebrae).

Statistical analyses were performed using IBM SPSS (version 19; SPSS, Chicago, IL). The primary end point (ie, treatment response) was analyzed using logistic regression, adjusting for the three factors specified in the minimization algorithm. The continuous secondary outcome measures were examined using analysis of covariance, adjusting for the baseline value of the measure together with the three minimization factors. Treatment effects are reported as adjusted point estimates together with the corresponding 95% CIs and *P* values. No interim analyses were performed.

The trial had ethics committee approval (United Kingdom 07/MRE00/59) and was conducted in accordance with the Declaration of Helsinki. It was registered with the European Union Drug Regulating Authorities Clinical Trials and ISRCTN databases.

## RESULTS

**Fig 1.**
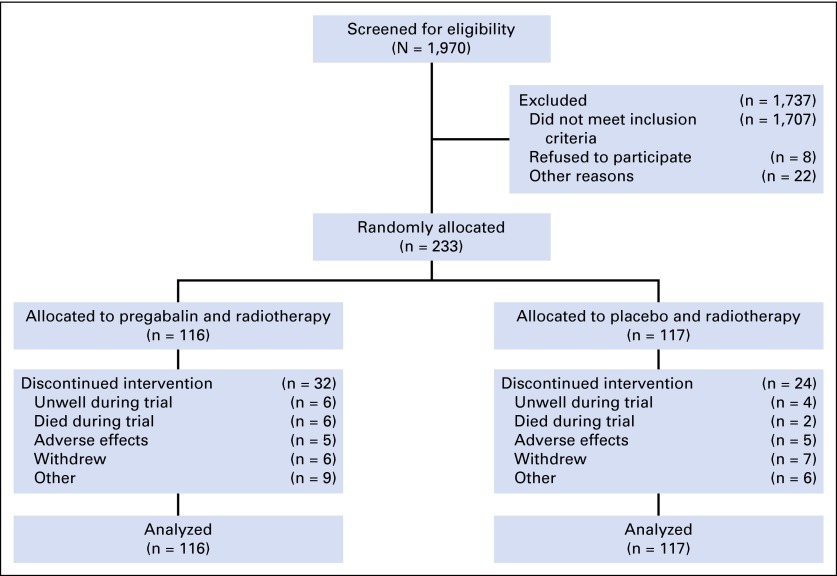
CONSORT diagram.

From August 13, 2008, to April 30, 2012, 233 patients were randomly assigned. The trial was stopped early (after 233 patients had been randomly assigned [target, n = 260]) on the basis of slow recruitment, evidenced by the fact that only one in eight patients screened was consented, as seen in the CONSORT diagram (Fig 1). The main reasons patients were unable to participate were poor renal function (19.0%), patient declined (8.7%), concomitant use of pregabalin or gabapentin (6.9%), or insufficient pain (4.5%).

Patient demographics are listed in Table 1. One hundred twenty-seven patients (55%) were male, and the mean age (± standard deviation) was 65.5 (± 10.97) and 63.7 (± 11.25) years for the placebo and pregabalin arms, respectively. Prostate (n = 88; 38%), breast (n = 77; 33%), and lung (n = 42; 18%) were the most common cancer types. Baseline characteristics were similar between the groups.

**Table 1.** Patient Demographic and Clinical Characteristics

Characteristic	Pregabalin Arm* (n = 116)		Placebo Arm (n = 117)	
	No.	%	No.	%
Age, years				
≤ 44	5	4.3	5	4.3
45-64	57	49.6	47	40.2
≥ 65	53	46.1	65	55.6
Male sex	59	50.9	71	60.7
Primary cancer type				
Bladder	1	0.9	4	3.4
Bone	1	0.9	0	0
Breast	41	35.3	36	30.8
GI	5	4.4	6	5.3
Renal	1	0.9	0	0
Larynx	1	0.9	0	0
Lung	23	19.8	19	16.2
Myeloma	0	0	1	0.9
Prostate	41	35.3	47	40.2
Skin	0	0	1	0.9
Unknown	2	1.7	3	2.6
Pain assessment at baseline				
BPI Intensity (0-40)				
Mean	18.63		18.68	
SD	7.51		6.58	
BPI Interference (0-70)				
Mean	39.90		36.48	
SD	16.30		14.54	
BPI Total (0-130)				
Mean	59.95		59.03	
SD	20.18		18.62	

Abbreviations: BPI, Brief Pain Inventory; SD, standard deviation.

* Data on age are missing for one patient in the pregabalin arm.

In the pregabalin arm, 45 patients (38.8%) achieved the primary end point, compared with 47 (40.2%) in placebo arm (adjusted odds ratio, 1.07; 95% CI, 0.63 to 1.81; *P* = .816). The observed absolute difference in response rates was 1.4% in favor of placebo (40.2% *v* 38.8%), with a 95% CI ranging from 13.9% in favor of placebo to 11.2% in favor of pregabalin. In the pregabalin group, 18 patients (15.5%) did not achieve the 2-point reduction in worst pain but rather remained on a stable or decreased opioid dose; 21(18.1%) had an increase in opioid dose, and 32 (27.6%) could not be assessed (assumed nonresponse). In the placebo group, 14 patients (11.9%) did not achieve the 2-point reduction in worst pain but instead remained on a stable or decreased opioid dose; 31 (26.5%) had an increase in opioid dose, and 25 (21.4%) could not be assessed (assumed nonresponse).

**Fig 2.**
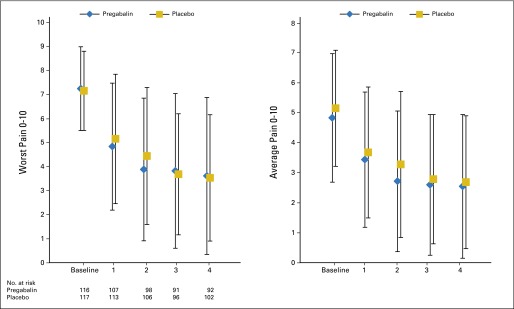
(A) Worst and (B) average pain per treatment arm from baseline to week 4 (by week). Adjusted difference mean (adjusted for three randomization stratification factors: fractionation regimen, cancer type, and site of bone metastasis): (A) −0.13 (95% CI, −1.02 to 0.75; *P* = .769) and (B) −0.52 (95% CI, −1.22 to 0.19; *P* = .150).

Figure 2A details the worst pain from baseline to end point (week 4) per trial arm. There were no statistically significant differences between trial arms. The adjusted difference in mean worst pain scores between trial arms was −0.13 (95% CI, −1.02 to 0.75; *P* = .769).

Figure 2B details the average pain from baseline to end point (week 4) per trial arm. There were no statistically significant differences between trial arms. The adjusted difference in mean average pain scores was −0.52 (95% CI, −1.22 to 0.19; *P* = .150).

Table 2 summarizes the BPI Intensity and Interference subscale, HADS, and EuroQol scores between trial arms. The adjusted mean difference between arms for BPI Intensity score was −0.7 (95% CI, −3.5 to 2.1; *P* = .606). The adjusted mean difference between trial arms for BPI Interference score was −1.8 (95% CI, −7.4 to 3.9; *P* = .537). There was a difference in HADS score between study arms (−1.1; 95% CI, −2.1 to 0.1; *P* = .031), but no difference in Euroqol score (*P* = .825).

**Table 2.** BPI, EuroQol, and HADS Scores Between Treatment Arms

Measure	Baseline			End Point (week 4)										
	No. of Patients	Score		No. of Patients	Score		Change		Difference			Adjusted Difference*		
		Mean	SD		Mean	SD	Mean	SD	Mean	95% CI	*P*	Mean	95% CI	*P*
BPI Intensity									−0.4	−3.3 to 2.4	.762	−0.7	−3.5 to 2.1	.606
Pregabalin	115	18.6	7.5	85	9.5	9.1	8.3	10.5						
Placebo	114	18.7	6.6	94	10.1	7.7	8.7	8.6						
BPI Interference									−1.4	−7.0 to 4.2	.629	−1.8	−7.4 to 3.9	.537
Pregabalin	110	37.9	16.3	80	24.2	19.3	9.9	18.4						
Placebo	113	36.5	14.5	90	26.0	18.3	11.3	18.0						
EuroQol									−0.6	−8.0 to 6.8	.868	−0.8	−8.2 to 6.6	.825
Pregabalin	114	54.6	19.6	86	60.1	24.1	3.8	24.1						
Placebo	113	55.3	19.6	92	60.2	23.0	4.4	25.5						
HADS									−1.0	−2.0 to −0.1	.039	−1.1	−2.1 to −0.1	.031
Pregabalin	105	19.8	3.3	82	20.8	3.0	−0.8	3.3						
Placebo	112	20.7	3.0	88	20.5	3.1	0.2	3.1						

Abbreviations: BPI, Brief Pain Inventory; HADS, Hospital Anxiety and Depression Scale; SD, standard deviation.

* Adjusted for three randomization stratification factors: fractionation regimen, cancer type, and site of bone metastasis.

Table 3 lists the serious and common adverse events (AEs) between trial arms. Fifty-one serious AEs were reported: 27 (53%) in the pregabalin arm and 24 (43%) in the placebo arm. Only one serious AE was thought to be related to pregabalin. The most common AE in the pregabalin arm was cognitive disturbance (23 events).

**Table 3.** Adverse Events

Adverse Event	No. (%)	
	Pregabalin Arm	Placebo Arm
Serious		
No. of events (n = 51)	27 (53)	24 (47)
Expected*	19 (37)	11 (22)
Likely related to IMP†	1 (2)	2 (4)
Common		
No. of events (n = 266)‡	183 (67)	83 (31)
Nausea	15 (6)	21 (8)
Cognitive disturbance	23 (9)	8 (3)
Vomiting	9 (3)	8 (3)
Fatigue	11 (4)	8 (3)
Pain	8 (3)	10 (4)

Abbreviation: IMP, investigational medicinal product.

* In keeping with underlying disease.

† In opinion of investigator.

‡ Most common events.

Table 4 lists breakthrough pain characteristics. These were similar between arms, with the exception of duration of breakthrough pain episodes, which was lower in the pregabalin arm (*P* = .037).

**Table 4.** Breakthrough Pain

Feature	Pregabalin Arm (n = 45)		Placebo Arm (n = 54)		P*
	No.	%	No.	%	
No. of episodes					.230
0-3	30	67	30	56	
4-6	6	13	12	22	
> 7	8	18	6	11	
Unknown	1	2	6	11	
Severity (0-10 NRS)					.175
0-3	19	46	15	29	
4-6	10	24	16	31	
> 7	12	29	20	39	
Duration of episode, minutes					.037
< 1	7	21.88	4	8.51	
1-15	17	53.13	19	40.43	
16-30	5	15.63	12	25.53	
31-60	1	3.13	5	10.64	
60-120	0	0.00	2	4.26	
> 120	2	6.25	5	10.64	
Time from onset to maximum intensity					.123
Unpredictable	11	34.38	11	22.92	
< 10 seconds	11	34.38	9	18.75	
10 seconds to 5 minutes	6	18.75	16	33.33	
6-30 minutes	4	12.50	10	20.83	
31-60 minutes	0	0.00	2	4.17	
Predictability					.657
Never	15	39.47	21	41.18	
Sometimes	15	39.47	16	31.37	
Often	0	0.00	3	5.88	
Almost always	2	5.26	7	13.73	
Always	6	15.79	4	7.84	
Use of analgesia					.266
Every time	8	21.05	8	16.00	
Most of the time	9	23.68	13	26.00	
Some of the time	6	15.79	16	32.00	
Hardly ever	9	23.68	6	12.00	
Never	6	15.79	7	14.00	

Abbreviation: NRS, numeric rating scale.

* Mann-Whitney test.

## DISCUSSION

This large clinical trial does not demonstrate an analgesic benefit from pregabalin in patients with CIBP receiving radiotherapy. Individual pain measures were similar across both arms, and the consistency of neutral primary and secondary end points supports that the study was sufficiently powered to detect a difference. Despite the trial under-recruiting, the primary result comfortably excludes the 20% advantage to pregabalin, which was the basis of the power calculation, and indeed, the 95% CIs were compatible with, at most, an 11.2% improvement in response rate, which would not be clinically meaningful.^18,19^

Of note, patients in the pregabalin arm experienced improvements in mood and reduced duration of breakthrough pain episodes. However, these must be considered in the context of the large CIs present and the established role of pregabalin for treating anxiety.

The current findings will have considerable implications for clinical practice, because pregabalin is being used increasingly in the setting of CIBP. Although basic science work was encouraging, with a possible therapeutic role for *α*2-*δ* calcium channel ligands in rodent models of CIBP, there had been limited clinical validation.^9^ A case report examined gabapentin in CIBP and suggested possible benefit.^14^ It was followed by a randomized trial of pregabalin versus placebo, which suggested potential value of pregabalin in CIBP, although firm conclusions could not be drawn.^15^ Another study suggested pregabalin in combination with mirtazapine may be beneficial; however, it advised additional trials.^16^ Nevertheless, despite the limited evidence, pregabalin is often prescribed for CIBP, with some reviews of CIBP management recommending the use of pregabalin if there is a neuropathic component to the pain.^17^ However, our findings do not support a role for pregabalin in CIBP, and we suggest that its clinical role in CIBP be reconsidered.

The challenges of translating laboratory findings clinically, in pain, include the difficulty in replicating multidimensional pain in animals and the subjectivity of testing in laboratory conditions.^23^ In clinical trials of patients with peripheral neuropathic pain, gabapentinoids have consistently demonstrated significant benefit.^24-26^ However, only a minority of patients achieved substantial pain relief, with many not responding. Patients with CIBP generally have multiple serious morbidities and a more complex array of factors contributing to their hypersensitivity, as a result of immunologic responses to the cancer. Their pain state is more complex and may be less readily reversible by pharmacologic interventions. Animal models may also not respond to therapeutic intervention either qualitatively or quantitatively in the same way as patients with CIBP. These and other reasons may have resulted in the lack of translation of our findings seen herein.

The trial had some limitations. All patients had metastatic cancer, and as such, many had different sites of pain. We focused on the area of pain corresponding to the radiotherapy site, and pain assessments were performed accordingly. Also, patients' overall conditions were deteriorating, in keeping with the advanced nature of their disease, and therefore, quality-of-life parameters could have changed. The end point measures were assessed 4 weeks after radiotherapy. It has been advocated that the optimal time for assessing response to radiotherapy for CIBP is 8 weeks, and we cannot be certain that additional differences would not have been evident if the trial had assessed end points at this time.^27^ However, although there may have been a greater number responding, the relative difference between the two arms would not be expected to change across a period from 4 to 8 weeks. The optimal dose of pregabalin in this setting of advanced cancer is not known, and again, it could be argued that higher doses or longer trial duration should have been used. Patients were, however, titrated to the maximum-tolerated dose, which resulted in 67% of patients achieving a dose of ≥ 300 mg daily for at least 3 weeks. These aspects have to be considered in the context of high attrition, which may have increased further with longer trial duration. We also acknowledge that patients who have CIBP with neuropathic features may be more likely to benefit from pregabalin than those without; however, this was outside the scope of our trial. In our trial, we looked at those patients referred to radiation oncology for consideration of palliative radiotherapy for uncontrolled CIBP. Clearly, patients treated successfully with pregabalin or gabapentin, because of CIBP with clinical neuropathic features, would not have reached radiation oncology and therefore screening in this study. Undertaking pain trials in in patients with cancer is challenging, but our trial succeeded by using a rigorous, well-designed protocol, a small number of centers, and focused patient follow-up.

Despite the neutral findings, we support the opinion of Hardy et al^28^ on the need for trials in patients with advanced cancer. Currently, the majority of symptom control practice is based on either historical anecdote or low-level evidence, and the lack of research is paradoxical to its importance.^29^ There is a need for well-designed clinical trials that either support or (as in our trial) refute practice. We have demonstrated that undertaking symptom control trials in patients with cancer is feasible.

In conclusion, our findings do not support the role of pregabalin in patients receiving radiotherapy for CIBP. Future trials examining pregabalin in CIBP with a neuropathic pain component would be of interest.
